# Commentary: A Breath of Fresh Air on the Mesenchyme: Impact of Impaired Mesenchymal Development on the Pathogenesis of Bronchopulmonary Dysplasia

**DOI:** 10.3389/fmed.2016.00013

**Published:** 2016-03-31

**Authors:** Eric S. White

**Affiliations:** ^1^Division of Pulmonary and Critical Care Medicine, Department of Internal Medicine, University of Michigan Medical School, Ann Arbor, MI, USA

**Keywords:** bronchopulmonary dysplasia, mesenchymal cells, fibrosis, lung diseases, embryonic development

Bronchopulmonary dysplasia (BPD) is a chronic lung disease of prematurity that is only now beginning to be understood at the molecular level. The review by Chao et al. (1) elegantly takes us through mouse and human lung development to identify possible mediators of the abnormal mesenchymal response characteristic of the disease. However, it quickly becomes clear that many aspects of BPD development seem to mimic chronic lung disease in adults. For example, secondary septa formation is a critical step in alveologenesis, with alveolar simplification in BPD thought to result from disordered septation. In the emphysematous adult lung, enlarged airspaces reminiscent of alveolar simplification are also seen; importantly, evidence suggests that many mediators thought to be important in BPD development are also important in COPD pathogenesis, such as wingless and int (Wnt) (2, 3), fibroblast growth factor (FGF) (4, 5), and sonic hedgehog (Shh) (6). Similarly, exuberant collagen and elastin deposition during alveologenesis bears remarkable similarity to the deposition of extracellular matrix proteins during adult lung fibrogenesis (7), although it is unclear whether the pattern and relative quantities of proteins are also similar. These observations, plus others, suggest a potential stereotypic lung injury response resulting in disrepair in adult lung and abnormal development in the neonate.

Despite the differences in etiology, both BPD and chronic adult lung injury are characterized by elevated expression and activity of transforming growth factor (TGF)-β, perhaps the most well-known profibrotic cytokine. Implicated in epithelial–mesenchymal transition (EMT) (8), myofibroblast differentiation (9), and epithelial apoptosis (10), TGF-β overexpression may be pathogenic for BPD, emphasizing the critical need for TGF-β regulation during fetal lung development (11). Similarly, in adult lung, TGF-β overexpression is maladaptive, resulting in fibrosis that has been likened to recapitulation of developmental programs (12). Understanding how dysregulated TGF-β activity perturbs both normal development and postnatal lung repair has yet to be determined, but side-by-side comparisons of the role of mesenchymal cells in lung development and disease will likely shed further insight.

An intriguing aspect of the review by Chao et al. is the supportive role of the lipofibroblasts (LIFs), the interstitial fibroblasts identified in rodent lungs containing cytoplasmic lipid droplets (13) thought to be important in alveolar epithelial cell surfactant production. Despite one manuscript showing the existence of LIFs in human lung (14), controversy still remains (15) about the existence of this cell in humans. While LIFs seem to contribute to secondary septation in developing rodent lungs through transfer of lipids to Type II alveolar epithelial cells (16), their role in the adult rodent lung (if any) remains unclear. To be certain if LIFs are developmentally important and potentially reparative mesenchymal cells of the lung, identifying them in human lung of any age would be of paramount importance. Similarly, it will be important to determine whether LIFs are truly a separate type of mesenchymal cell or whether they are simply interstitial fibroblasts that uptake lipid droplets for transfer to alveolar epithelium. Currently, there are few known markers of LIFs, such as platelet-derived growth factor receptor (PDGFR)-α and peroxisome proliferator-activated receptor (PPAR)-γ (17), but these are not specific for LIFs. More work in this area will obviously be necessary.

Mesenchymal–epithelial crosstalk, as described above for LIFs and type II alveolar epithelial cells, is obviously important for lung development, as nicely illustrated by Chao and colleagues (1). However, less is known about the contribution of the endothelium, and more specifically mesenchymal–endothelial interactions, in the development of chronic lung diseases, including BPD. Clearly, the transformation of a double vasculature to a single capillary system in primary alveolar septae is integral to gas exchange in the developing lung, as pointed out by Chao and colleagues. The development of this vasculature occurs through both angiogenesis and vasculogenesis. Along the same line, angiogenesis and vasculogenesis appear to be important in the development of certain adult chronic lung diseases, such as COPD, asthma, and idiopathic pulmonary fibrosis (IPF) (18–20). Indeed, nintedanib, a tyrosine kinase inhibitor that blocks vascular endothelial growth factor receptor, FGF receptor, and PDGFR, has recently gained approval around the world for treatment of IPF because of its effects on slowing the rate of decline of lung function (21). It is not yet known whether the salutary effect of nintedanib in IPF is related to its inhibitory effects on angiogenesis.

We have certainly learned much about BPD pathogenesis through elegant mouse modeling and human pathologic studies. However, there is still much to learn; murine studies, while informative, cannot take the place of knowledge generated in human samples of disease. Moreover, although mouse lung development occurs in a stereotypic fashion which has been well characterized, it is not proven that human lung development occurs entirely in the same way. Thus, better methods of studying human lung development need to be created and validated, with findings hopefully informing our understanding of chronic adult lung diseases as well.

As alluded to by Chao and colleagues (1), the incidence of BPD and its long-term effects appears to be on the rise as more premature infants survive owing to advances in supportive care. There are no definitive therapies for these patients. Just as research into the lung mesenchyme will surely enlighten our understanding of BPD, researchers of all chronic lung diseases are likely to gain better insights into disease pathogenesis and potentially pave the way for future therapies in numerous diseases. Impaired mesenchymal development and responses – it is not just for BPD.

## Author Contributions

The author confirms being the sole contributor of this work and approved it for publication.

## Conflict of Interest Statement

The author declares that the research was conducted in the absence of any commercial or financial relationships that could be construed as a potential conflict of interest.
